# Motives for using social networking sites: a uses & gratifications perspective amongst people with eating disorder symptoms

**DOI:** 10.1186/s40337-023-00946-1

**Published:** 2023-12-19

**Authors:** Alexandra E. Dingemans, Jolanda Veldhuis, Irene Lähde

**Affiliations:** 1 Rivierduinen Eating Disorders Ursula, P.O. Box 405, 2300 AK Leiden, The Netherlands; 2 Department of Communication Science, Vrije Universiteit Amsterdam, Amsterdam, The Netherlands

**Keywords:** Social networking sites, Eating disorders, Uses and gratifications theory, Readiness to change

## Abstract

Studies investigating motives for social networking sites (SNS) use amongst people with eating disorder (ED) symptoms are scarce. The uses and gratifications theory states that people actively select media content to gratify their individual needs and this may help to explain why individuals with eating disorder symptoms choose to expose themselves to specific media content and how they choose to respond. Some evidence exists that readiness to change may be a factor related to SNS use patterns in this population. The aim of the present study was to investigate the potential relationship between readiness to change ED cognitions/behaviors and SNS use motives in individuals with current or past ED symptoms, employing a uses and gratifications theory perspective. Participants were recruited from ED recovery websites; 103 individuals (16–55 years old) provided complete data. As hypothesized, ED symptom severity was inversely associated with readiness to change and time spent on (specific) SNS was not associated with ED symptom severity. The results of hierarchical regression analyses further suggested that if individuals felt less capable of overcoming their dysfunctional cognitions/behaviors about their body/eating (i.e., were less ready to change), they were more likely to be motivated to use SNSs for the purpose of impressing others and reputation management. The clinical implication is that high amounts of SNS use may not necessarily be harmful for people with EDs or recovering from EDs, whereas using SNSs for the purposes of impression management could potentially be related to being less ready to recover.

**Supplementary Information:**

The online version contains supplementary material available at 10.1186/s40337-023-00946-1.

## Abstract (plain language summary)

People who feel they are capable of overcoming their dysfunctional cognitions and/or behaviours about their body and/or eating, are less likely to use social networking sites for impression management purposes. Motives for social media use like popularity, self-presentation, and social expectations may potentially hinder recovery form an eating disorder. Clinicians should discuss motives of SNS use with their clients, rather than total amount of screen time, to help prevent any negative media effects.

**Supplementary Information:**

The online version contains supplementary material available at 10.1186/s40337-023-00946-1.

## Background

Social networking sites (SNSs) have become a central part of everyday life and being active on these sites has gained popularity as a common pastime for many people [36]. Individuals spend many hours each day creating personal profiles, interacting with like-minded people and sharing their thoughts, feelings, and insights online. There were an estimated 3.6 billion SNS users worldwide in 2020, which represented approximately half of the world's population [44]. Given that SNSs are highly visual platforms and contain large amounts of thin-ideal and appearance-focused content, many studies found that SNS use (like the traditional media images in magazines and newspapers [30]) was associated with body image concerns and disordered eating in the general population (in both women and men) [12, 25, 50]. Studies investigating SNS use amongst people with clinical levels of eating pathology are generally scarce. More research into the SNS use in people with ED symptoms is important since the onset of EDs usually occurs in adolescence or young adulthood [34], when SNS use tends to be high [28].

Perloff [36] states in his translation model that how one uses social media is depended on specific predisposing individual characteristics, like a thin-ideal internalisation or the centrality of appearance to self-worth and on particular gratifications one seeks [36]. He suggested that more vulnerable people i.e., people with lower self-esteem and lower body satisfaction, are more likely to use SNSs to seek reassurance, procrastinate, escape from their problems, and reduce loneliness. Spending time on specific SNS can set in motion a set of psychological processes such as social comparisons or identification, which in turn can increase body dissatisfaction, negative affect or the onset of an eating disorder. Time spent on SNS does not seem to differ much between those with and without an eating disorder, however, the first group appeared to devote more online time to eating, weight and body related content than the latter [4]. Moreover, individuals with an eating disorder seemed to spent more time on forums/blogs where the ideas, opinions and feedback of peers play a large role [36], and less time on information, news and shopping sites than healthy controls. In other words, those with an eating disorder tend to use SNS in a different way than those without. Overall, the online world of people with ED was dominated with a more narrow focus on topics dominating their ED world in comparison to healthy controls [4].

In popular media, newspaper articles about SNSs “causing” eating disorders (EDs) have been abundant e.g. [3, 20]. However, what these articles usually tend to neglect is that SNSs can also have positive consequences [42, 50]. Being part of a social network provides a sense of belonging, recognition and self-worth (van den [49]. Importantly, this social compatibility not only holds true for SNSs that glorify eating disorders, but also for those that promote recovery. These latter sites promote ED recovery through allowing opportunities to share experiences and emotions with others who have been through similar journeys [5]. Therefore, it may be argued that it is not only the total amount of time spent on specific SNS that is relevant in the context of EDs [25, 54], but also the specific motives to use SNS by people with ED symptoms [36, 39].

The uses and gratifications theory [26] might be suited to exploring the motives why people with body image or eating concerns may engage in online activities. The uses and gratifications theory is a user-centered approach that focuses on how people use media for their own personal uses and gratification. This theory emphasizes motives and the self-perceived needs of audience members. In other words, this theory aims to explain why people actively select media content to gratify their individual needs [29] and may help to explain why individuals with eating disorder symptoms choose to expose themselves to specific media content and how they to respond behaviourally, emotionally, and cognitively to this content [14, 24]. They appear to use specific media and specific content to derive specific gratifications such as diversion, relaxation, entertainment, informative, escape or as a source of appearance standards [46]. For example, some may seek content related to fitspiration or thinspiration to lose more weight whereas others seek information on how to reach out for help provided by recovered expert patients. Such an approach in differentiating why and how people use social media ties in with current perspectives in the field of social media effects research, calling for a more nuanced investigation of social media use and more careful interpretation of its effects that cannot uniformly be attributed to all users. More specifically, it seems important to further explore how social media content, receiver and sender characteristics play a role in guiding the impact of social media, instead of only the time spent (actively or passively) online (e.g., [31, 48]. From this theorizing, it can also explain why some people with ED symptomatology turn to social media content focusing on weight and eating or, alternatively, some seek support for their recovery [4, 21]. Put differently, this uses and gratifications perspective might also explain how motives to use SNS change over time as well as the SNS that will be visited. For example, in the first stages of the illness one may search for information to lose weight whereas in later stages one may search for ways to recover. In the context of eating disorders, these motivations for using social networking sites might be linked to attitudes that have been defined as readiness to change. In other words, one's preparedness to make changes, encompassing both the desire and ability to address and modify symptoms of an eating disorder [16].

The ego syntonic nature of eating disorders, especially in those suffering from anorexia nervosa, may inhibit the readiness to change and is a key component in the maintenance of the disorder [22]. The ambivalence toward recovery may be explained by the fact that the disease gives sufferers a sense of control, however, when the disease begins to overtake their thoughts and behaviour, it begins to divest them of control [22]. Some tentative evidence pointed to the readiness to change their ED behaviours and cognitions as a factor influencing SNS use motives of those suffering from ED. Individuals who were less ready to change appear to be using SNSs for seeking information about diets and exercise, whereas readier individuals were motivated by wanting to support others emotionally [41]. Smahelova and colleagues (2019) speculated that as people become more capable of changing, and change their attitude towards their ED, they also change their attitudes towards online content and thus are driven by different motives than those who are not ready to change their disordered behaviours. However, this qualitative study did not include quantitative measures of readiness to change. Thereto, the present study tries to fill the gaps in the current body of literature by investigating the motives for SNS use among people with a clinical level of eating disorder symptoms in relation to the severity of their ED symptomatology and their readiness to change their current ED cognitions and behaviours.

The general aim of the present study was to investigate the potential relationship between readiness to change ED cognitions/behaviours and SNS use motives in individuals with current or past ED symptoms, employing a uses and gratifications theory perspective. Our first research question was whether SNS use in general is related to ED severity. Based on a previous studies [4, 25] which concluded that time spent on SNS did not differ between those with and without an eating disorder, we hypothesized that (H1) SNS use was not associated with higher symptom severity (i.e. ED symptom severity, body satisfaction and self-esteem). Our second question was whether ED severity was related to readiness to change. In line with previous studies [43], we hypothesized that (H2) higher readiness to change was associated with lower levels of ED symptoms. Previous studies showed that readiness to change is linked to symptom improvement [19, 43]. Research question 1 and 2 were added to confirm that the results of the current study are in line with past research. Our third research question was whether specific motives to use SNS were related to severity of ED psychopathology and readiness to change. Given that most uses and gratifications studies regarding SNS use have used healthy student samples, it was not clear which motives would be relevant for the present clinical sample. Given the evidence that more social interaction and greater self-expression predicted likelihood of recovery [5], and that recovering participants reported using specific SNS for documenting their recovery journey, seeking positive emotions, and supporting others in their recovery [10, 42], it was expected that (H3) higher readiness to change and less severe ED symptoms would be associated with higher ratings of these motives. However, motives like avoiding loneliness, relieving boredom, and escaping from problems are likely to motivate SNS use amongst people more vulnerable to body image issues [36]. Also, passively following of others was related to more ED psychopathology and reputation management on SNS was associated with appearance anxiety [36]. Given these findings, it was expected that (H4) lower readiness to change and more severe ED symptoms would be associated with higher endorsement of these latter motives.

## Methods

### Participants

To be included in the study, participants had to be at least 16 years of age, reporting current or historical ED symptoms, and own at least one SNS account. In total, 163 participants provided informed consent, but only 146 met the inclusion criteria (those who did not, were automatically directed to the end of the survey). One hundred and three complete survey responses were collected from visitors of a large e-community for individuals with eating problems or disorders (i.e. Proud2Bme) as well as from three pro-recovery patient organizations supporting people with EDs. Proud2Bme is a Dutch e-community for people with eating related problems with a focus on self-acceptance and recovery and is part of GGZ Rivierduinen Eating Disorders Ursula, Leiden, The Netherlands. The ad text stated that the aim was to investigate what motivates people with (a history of) eating disorder problems to use social media. The rationale behind not having stricter inclusion criteria (e.g. requiring a diagnosis, or scoring above a clinical cut-off) was to obtain a diverse, representative sample to improve the generalizability of the results(see Table 1. for descriptive information regarding the demographic and clinical variables).

**Table 1:** Means and Standard Deviations of Demographical and Clinical Variables of Complete Response Sets

Age (in years), *n*, *M* (SD)	143	26.1 (8.10)
Global EDE-Q (range 0–6), *n*, *M* (SD)	118	3.43 (1.58)
Body Satisfaction, *n*, *M* (SD)	115	3.73 (2.06)
Self-Esteem (range 0–5), *n*, *M* (SD)	115	1.94 (1.1)
Readiness to Change (range 0–10), *n*, *M* (SD)	114	6.81 (2.24)
BMI, *n*, *M*(SD)	96	19.33 (3.4)
Frequency objective binge episodes, past 28 days, *n*, *M* (SD)	124	2.59 (6.17)
Frequency objective binge episodes, past 28 days*, *n*, *M* (SD)	33	8.64 (8.34)
Frequency self-induced vomiting, past 28 days,*n*, *M* (SD)	124	2.21 (5.82)
Frequency self-induced vomiting, past 28 days*, *n*, *M* (SD)	25	9.24 (9.05)
Frequency laxative misuse, past 28 days, *n*, *M* (SD)	124	1.57 (8.45)
Frequency laxative misuse, past 28 days*, *n*, *M* (SD)	11	10.45 (24.5)
Frequency excessive exercising, past 28 days, *n*, *M* (SD)	124	10.1 (12.13)
Frequency excessive exercising, past 28 days*, *n*, *M* (SD)	64	15.5 (12.42)
Gender, *n*(%)		
Male	4	3%
Female	139	97%
*Living situation, n(%)*		
Living with parents	57	40%
Living alone	45	32%
Living with partner/children	28	20%
Student housing	9	6%
Other/Prefer not to say	4	3%
Highest educational level, *n*(%)		
*Primary*	41	28%
Vocational	46	36%
Higher professional	25	17%
University	31	21%
*Socio-economic status, n(%)*		
Student	56	39%
Employed full-time	23	16%
Employed part-time	25	18%
Unemployed/homemaker	6	5%
Disability support/unable to work	28	20%
Other/Prefer not to say	5	4%
Country of birth		
Netherlands	131	92%
Belgium	10	7%
Other European country	1	1%
Other non-European country	1	1%

Rows with * refer to the respondents who reported doing these behaviors at least once in the past 28 days (values of zero excluded from the corresponding means and standard deviations). EDE-Q = Eating Disorder Examination-Questionnaire; BMI = Body Mass Index

## Design and procedure

A cross-sectional research design was applied. An online survey, taking approximately 20 min to complete, was created on a secure SurveyMonkey account. Access to the survey was provided via a link on the websites from 27 May 2020 until 1 August 2020. Following study information and informed consent, participants were asked to complete screening questions relating to inclusion/exclusion criteria. If they did not meet the criteria, they were directed to the end of the survey. Those who completed the study, were offered the option to enter their email address to participate in a raffle to win one of ten bol.com vouchers (10 euros in value). Email addresses were the only identifying information collected, and these were removed from the dataset and kept separately and securely from the rest of the data. The email addresses were only used to contact winners once the study had ended. The Psychology Research Ethics Committee of the Leiden University approved this study.

## Measures

### Demographics

Participants were asked to indicate their gender (male, female, other); age (in years); living situation (living with parents/family, living alone, living with partner/children, student housing, other/prefer not to say); highest educational level achieved (answering options: primary, vocational, higher professional, university), employment status (student, homemaker, employed fulltime of part-time, unemployed, disability/unable to work, other)), civil status (single, co-habiting/married/divorced),, and country of birth (Netherlands, Morocco, Antilles, Turkey, Suriname, other).

### Eating disorder history

To further describe the sample, participants were asked about current/past ED treatment, and the duration of their symptoms.

### Eating disorder symptom severity

The Eating Disorder Examination Questionnaire (EDE-Q 6.0) [11] consists of 28 items assessing the frequency and severity of ED symptoms over the past 28 days, using a seven-point Likert-scale ranging from 0 to 6. These include six questions assessing the frequency of core ED behaviors (binge eating, self-induced vomiting, laxative use, excessive exercise, fasting, use of diuretics), and 22 questions relating to psychological features (dietary restraint, eating concern, weight concern, and shape concern). A global score of eating psychopathology was obtained by averaging the 22 items relating to psychological features, with higher scores reflecting more severe ED psychopathology [1]. Internal consistency was high for those 22 items (α = 0.96). The questionnaire also asked about weight and height.

Body mass index (BMI) was computed by dividing the self-reported weight (in kilograms) by the squared self-reported height (in meters).

### Body (Dis)satisfaction

Participants were asked to rate their (dis)satisfaction with their appearance, the shape and size of their body, their weight, and their physical attractiveness compared to others, on a scale from 1 (very dissatisfied) to 10 (very satisfied). The total score was obtained by computing the mean of the four responses [51]. Internal consistency was high (α = 0.92).

### Self-esteem

Participants were asked to complete the Single-Item Self-Esteem Scale (SISE) [38], a one-item measure of global self-esteem (“I have high self-esteem.”). The answer was provided on a 5-point Likert scale, ranging from 1 (not at all true of me) to 5 (very true of me).

### Readiness to change

The Eating Disorder Readiness Ruler (ED-RR) [43] is an 18-items questionnaire examining readiness to change in nine ED symptom domains paralleling those in the EDE-Q. In the present study only the first section was used (items 1 to 9) measuring readiness to change over the past 28 days. Responses ranged from 1 to 10 (1–2 = not at all ready to change, 3–5 = unsure, 6–8 = ready to change, 9–10 = actively changing already). There was also the option of responding “not applicable”, if the behaviour or cognition in question was not relevant to the participant. A mean score for readiness to change was obtained by computing a summed score and then dividing the sum by the number of items completed for each participant so symptom domains that were not applicable to the participant were not included in the calculation[43]. A higher score reflected a higher readiness to change. Cronbach’s alpha was only calculated for the items that had more than 50% endorsement: dietary restraint, shape concern, weight concern, and excessive exercise. Internal consistency was good for these four items (α = 0.84).

### General internet and social media use

Participants were asked about their amount of average daily internet and social media use [32]. The options were: never/almost never, less than an hour a day, between one and two hours a day, between two and three hours a day, or more than four hours a day.

### Specific SNS use

For the present study SNS was defined as applications and websites that enable users to create and share content with networks (i.e., friends, followers, etc.) they construct for themselves [37]. The focus was on general-purpose sites, and therefore specialized instant messaging platforms (e.g. WhatsApp), career services (e.g. LinkedIn), and dating applications (e.g. Tinder) were excluded. Similarly, specialized pro-ED (pro-ana/pro-mia), and mHealth/eHealth and fitness/diet mobile applications were beyond the scope of the present study. The SNSs included were the most popular general-purpose ones in the Netherlands [44]: Facebook, Instagram, Pinterest, Twitter, YouTube, Snapchat, and Tumblr. Given the recent popularity of the TikTok platform [44], this was added as one of the options. Participants were asked to indicate the amount of time they spent daily on each of the platforms using two drop-down menus: one for hours per day and another for minutes per day. These were combined into overall minutes per day. Total daily SNS use was computed by adding the responses for each platform together [54].

### Motives for SNS use

Participants were asked to express their agreement/ disagreement with 75 statements obtained from the uses and gratifications literature (for the origin of the items and specific references: see Additional file 1: Table 1). These statements measured the following 16 motives: enjoyment (three items), social interaction (eight items), passing time (five items), surveillance (seven items), information-seeking (five items), information-sharing (three items), relaxation (four items), avoiding loneliness (three items), escapism (three items), support (four items), self-documentation (four items), connecting with similar others (seven items), self-expression (five items), social pressure (six items), popularity (five items), and self-presentation (three items). These motives were selected because of their relevance in several different studies and applicability to several SNS platforms. The statements were on a five-point Likert-type scale anchored by “Strongly disagree” (1) to “Strongly agree” (5). The scores of the 16 variables were obtained by computing the median of the individual items measuring the variables. For example, a participant’s score for “enjoyment” was determined by obtaining the median of the responses to the three items measuring enjoyment: “Because it is fun”, “Because it is enjoyable”, “Because it is entertaining”.

### Data analysis

The Statistical Package for the Social Sciences (SPSS) version 26.0 was used for all analyses. Since none of the variables were normally distributed Spearman rank-order correlations were computed with bootstrapping (1000 samples) to investigate the association between the variables.

Regarding the motives for SNS use, an exploratory factor analysis was conducted to reveal any latent variables influencing the covariance of the motives. Initially, the factorability of the motives was examined. The Kaiser–Meyer–Olkin measure of sampling adequacy was 0.761, well above the recommended value of 0.6, and Bartlett’s test of sphericity was significant, χ^2^ (120) = 590.49, *p* < 0.001. As such, factor analysis was deemed to be suitable with all 16 motives. Given the non-normality of the motive variables, the robust method of principal axis factor analysis was selected [8]. Costello and Osborne [8] recommend that oblique rotations should be selected over orthogonal rotations if the variables are even somewhat correlated (see correlation matrix in Additional file 3: Table 3). As such, a promax rotation was applied. The criteria for a factor to be retained were an eigenvalue of at least one, and at least three items meeting primary loadings of at least 0.5 with no secondary loadings above 0.4 (as per [35].

Following factor analysis and identification of the factors, factor scores were created for the purposes of using these in multiple regression analyses. Factor scores were computed by calculating the mean of all the items that measured the motives that loaded above 0.4 on a specific factor.^1^ It was decided that it was not necessary to standardize these values, given that all the items were on the same Likert scale.

Factors were treated as outcome variables in a series of hierarchical multiple regression analyses, to investigate how ED severity, readiness to change, self-esteem, and body satisfaction, and total amount of daily SNS use (in minutes) predict the motives driving SNS use. Given previous evidence that younger people are heavier SNS users than older people [27], the decision was made to control for age. The assumption of collinearity was met if VIF values were below ten and tolerance values were greater than 0.1. The assumption of independence of errors was met if Durbin-Watson values were greater than one and less than three.

## Results

### Sample characteristics

No significant differences in the demographic variables were found between the participants who completed all the data and those who did not. With one exception, there was a significant association between living situation and completing the data: χ^2^(5) = 11.23, *p* = 0.05. Those who did not provide complete data were more likely to live alone.

In terms of treatment for ED symptoms, 68 (47.6%) reported that they were currently receiving treatment, 60 (42%) reported that they have had received treatment in the past, and 15 (10.5%) reported they had never received any treatment. Ninety percent of the sample has or had a formal diagnosis. Those who reported current ED symptoms (*n* = 83) reported a mean illness duration of 9.12 years (*SD* = 4.90), and 13 (8.9%) reported having had symptoms for more than 20 years. The mean ED psychopathology severity score, as measured by the global EDE-Q score (*M* = 3.45), was higher than the clinical cut-off of 2.17 [9]. Table 1. shows the clinical characteristics of the sample.

As for their internet and SNS use, most respondents (99%) reported to use the internet at least for one hour a day. Seventeen percent reported using SNS less than one hour, 43% 1 to 2 h, 31% 2 to 3 h and 9% more than 4 h a day. Facebook was the SNS with most users, but Instagram was the most used overall (see Table 2.). The mean total SNS use was 176.6 min (*SD* = 120.57), or 2.94 h.

**Table 2:** Respondents Reporting Daily SNS Use Per Platform, and the Mean Daily Use (Minutes)

SNS	Frequency	%	Mean	SD
Facebook	88	85.44	45.00	38.45
Instagram	85	82.52	69.29	47.52
YouTube	81	78.64	59.14	50.76
Snapchat	43	41.75	27.79	27.52
Pinterest	34	33.01	24.12	18.65
TikTok	15	14.56	69.00	70.89
Twitter	10	9.71	34.50	33.87
Tumblr	6	5.83	25.83	20.84

Percentages refer to complete data (*n* = 103). Means and standard deviations refer to the respondents reporting to use the SNS in question (those responding “not applicable” were not included in the calculations)

### SNS Use and ED Severity

Regarding H1, in line with our expectations, SNS use was unrelated to ED symptom severity (i.e. ED symptom severity (EDE-Q global, *r* = -− 0.07), body satisfaction (BDQ, *r* = -− 0.04), self-esteem (*r* = -0.05).

### ED symptom severity and readiness to change

As hypothesized (H2), ED symptom severity had a moderate negative association with the global score for readiness to change (*r*_s_ = -− 0.47, *p* < 0.001, bootstrapped CI [− 0.61 − -− 0.29]). In line with expectations, ED symptom severity had a strong negative association with body satisfaction (*r*_s_ = -− 0.72, *p* < 0.001, bootstrapped CI [− 0.82 − -− 0.57]), and a moderate negative association with self-esteem (*r*_s_ = -− 0.46, *p* < 0.001, bootstrapped CI [− 0.62 − -0.30]).

### Motives to use SNS, ED symptom severity and Readiness to change

**Fig. 1:**
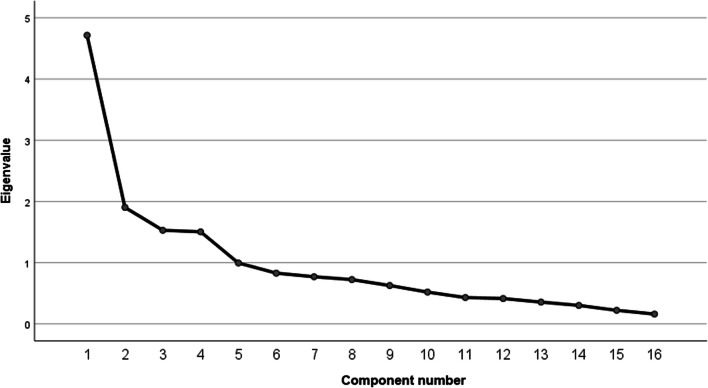
Scree plot factor analysis motives to use social network sites

For testing H3 and H4, we started with an overview of the frequency of the mentioned motives. The most endorsed motives overall (statements most agreed with; means above three and modes and medians of four) were enjoyment, social interaction, information-seeking, habitual use, and surveillance (see Table 3). The least relevant (means below two, modes and medians of one) were popularity and self-presentation. The factor-analysis provided four factors with an eigenvalue above one (see Fig. 1). The four-factor solution explained 60.32% of the variance. The internal consistency of the items making up each factor was high. Information-sharing (0.66), connecting with similar others (0.80), and support (0.88) had large positive loadings on factor 1. This factor appears to describe supporting others, providing helpful information to others, as well as creating a community of similar others. As such, this factor was called “Community” (Cronbach’s alpha = 0.92; Eigenvalue = 4.71; Variance explained = 29.46%). Self-presentation (0.98), popularity (0.87), and social pressure (0.40) had positive loadings on factor 2. Given that this factor seems to be about the importance of social expectations, and how the user appears to an audience, it was called “Impression Management”(Cronbach’s alpha = 0.90; Eigenvalue = 1.9; Variance explained = 11.9%). Surveillance (0.42), passing time (0.79), escapism (0.61), and avoiding loneliness (0.65) loaded positively on factor 3, which suggests that this factor (subsequently named “Passive Use”) is concerned with passive, solitary use, as well as avoidance of negative emotions (Cronbach’s alpha = 0.91; Eigenvalue = 1.53; Variance explained = 9.56%). Self-documentation (0.57), self-expression (0.46), enjoyment (0.60), and relaxation (0.54) loaded positively on factor 4. This factor appears to describe the active seeking of genuine positive emotions, as well as the sharing and expressing of one’s authentic self. As such, factor 4 was called “Positive Use” (Cronbach’s alpha = 0.9; Eigenvalue = 1.51; Variance explained = 9.4%) (see also Additional file 3 and 4: Table 3 and 4).

**Table 3:** Ratings of SNS Use Motives (*n* = 103)

	Mean	SD	Mode	Median	α
Enjoyment	4.05	0.73	4	4	0.83
Social Interaction	3.79	1.06	4	4	0.89
Information-Seeking	3.74	1.09	4	4	0.79
Passing Time	3.62	1.12	4	4	0.78
Surveillance	3.57	1.18	4	4	0.94
Relaxation	3.13	0.95	3	3	0.88
Avoiding Loneliness	2.89	1.39	4	3	0.84
Escapism	2.88	1.37	4	3	0.79
Support	2.75	1.24	4	3	0.82
Self-Documentation	2.74	1.24	4	3	0.83
Information-Sharing	2.7	1.41	1	3	0.77
Connecting with Similar Others	2.6	1.35	1	3	0.91
Self-Expression	2.55	1.31	1	3	0.89
Social Pressure	2.48	1.37	1	2	0.84
Popularity	1.74	1.12	1	1	0.89
Self-Presentation	1.6	1.03	1	1	0.75

Scale used: 1 = strongly disagree, 2 = disagree, 3 = neither agree nor disagree, 4 = agree, and 5 = strongly agree

**Table 4:** Hierarchical Regression Results

Predictor variables	Community β	Impression Management β	Passive use β	Positive Use β
*Block 1*				
Age	0.08	− 0.21*	− 0.15	− 0.06
Δ*R*^*2*^	0.00	0.07	0.04	0.02
*Block 2*				
Global EDE-Q	0.07	0.08	0.12	0.20
Body Satisfaction	− 0.03	0.29*	0.14	0.21
Self-Esteem	0.06	0.03	− 0.25	0.16
Readiness to Change	− 0.03	− 0.27*	− 0.04	0.03
Δ*R*^*2*^	0.02	0.06	0.09	0.05
Block 3				
Total Daily SNS Use (Minutes)	0.37***	0.24*	0.29**	0.36***
Δ*R*^*2*^	0.13	0.05	0.08	0.12
*R*^2^	0.15	0.19	0.21	0.19
Adjusted *R*^2^	0.10	0.14	0.16	0.14

Figures are standardized beta coefficients in the last step of hierarchical regression

^*^p < 0.05, **p < 0.01, ***p < 0.001. EDE-Q = Eating Disorder Examination-Questionnaire; SNS = social networking site

Following the uses and gratification principles, hierarchical regression analyses with the four motive dimensions (Community, Impression Management, Passive Use, and Positive Use) treated as outcome variables showed that total SNS use was significantly associated with all the motive dimensions (see Table 4). The assumptions of collinearity (VIF values were below ten) and tolerance values (greater than 0.1) were met. The assumption of independence of errors was met since Durbin-Watson values were greater than one and less than three (see Additional file 5 and 6: Table 5 and 6). This outcome suggested that people who are highly motivated to use SNSs for a specific reason are also more likely to use SNSs in higher amounts. However, in further testing our predictions in H3 and H4, the hierarchical regression analyses showed that the clinical variables body satisfaction and readiness to change emerged as significant predictors of the Impression Management factor. In other words, participants who were less ready to change their ED behaviors and/or cognitions, and were nevertheless more satisfied with their bodies, were more likely to use SNSs to create an identity for an audience, to gain popularity, and to be driven by social pressures and expectations. Contrary to our expectations, the other three motives dimensions were not associated with the clinical variables (ED symptom severity, readiness to change, body satisfaction, self-esteem).

## Discussion

This study aimed to explore SNS use as well as motives behind SNS use, and their potential relationships with ED psychopathology and readiness to change, among participants reporting current or past ED symptoms. Regarding our first hypothesis (H1), it was expected that ED psychopathology was not associated with the amounts of daily SNS use. Indeed, the results of our current study supported this hypothesis. It may be hypothesized that the ED psychopathology of the participants is determining the specific type of SNS content they seek, rather than their time spent on SNSs. This assumption is in line with the suggestion by Holland and Tiggemann [25] that engagement with appearance-related SNS content influences body image concerns, rather than general (time spent on) SNS use. Indeed, Griffiths et al. [23] also found that more frequent exposures to specifically fitspiration (idealized depictions of fit, lean, toned bodies) and thinspiration (idealized depictions of extremely thin bodies) content was associated with experiencing more severe ED symptoms. Furthermore, not finding such an association between the general time spent on SNS and ED psychopathology also aligns with recent developments in the field of social media effects research. Scholars in this field call for a more nuanced approach in the investigation and interpretation of social media’s impact. That is, we should to go further than just accounting for the time spent (actively or passively) online, but rather take into account the specifics of social media content, its creators, and its users to further clarify social media’s differential impact (e.g., cf. reasoning in [31, 48].

The second research question concerned readiness to change and its relationships with ED symptom severity, body satisfaction, and self-esteem. As predicted (H2), readiness to change was negatively associated with ED symptom severity and body dissatisfaction. This outcome is consistent with the findings by Geller and colleagues [16, 17] who found that individuals actively working on change reported less severe ED symptoms and were more satisfied with their body and shape. In another study of the same research group [18], it was also suggested that although high levels of psychological distress may motivate change in general, active change of individual behaviors and cognitions can only occur if the distress levels are reduced (to a level that people can handle). No significant correlation between readiness to change and self-esteem was found.

Then, this study further aimed to explore the motives for SNS use that would be relevant to people with ED symptoms. From the theoretical principles underlying selective self-presentation [15], we expected that SNSs would provide many opportunities for managing and enhancing one’s identity to build social capital [33]. Yet, this seems not to be leading motive for people with current or past ED symptomatology. Much like people in the general population [53], individuals with current or past ED symptoms also used SNS the most for enjoyment, social interaction, information-seeking, and passing time. Surprisingly, social pressure, popularity, and self-presentation were the least endorsed motives in our sample. However, lurkers, typically members of an online community who observe, but do not participate, comprise the majority of all users in online communities [45]. It could therefore be that the majority of individuals with an eating problem do not actively participate on social media.

The third research question that was central to our study, pertained to whether motives to use SNS were related to severity of psychopathology and readiness to change. The factor analysis in our sample revealed four motive dimensions: Impression Management, Community, Passive Use, and Positive Use. Social interaction and information-seeking did not load highly on any of the identified dimensions. However, this may be due to the universality of these motives, demonstrated by their importance in general population samples [2, 53]. Indeed, these were generally highly endorsed by the present sample.

Next, we will reflect on our third hypothesis, stating that a higher readiness to change and less severe symptomatology would relate to higher scores on using SNS for the motives of support an positive emotions (H3; [10, 42]. We could argue that the motive domains of Community and Positive Use we found in our study cover the description of H3. However, the analyses did not confirm our third hypothesis (H3). We found that the Community factor (relating to sharing, connecting, and supporting others) was not related to any of the clinical variables, which was an unexpected finding, given previous evidence of pro-social SNS motivations amongst people recovering from EDs [10, 41]. This may be explained by the fact that we recruited participants from pro-recovery communities. Therefore, pro-ED individuals are probably underrepresented in our sample. Overall, pro-ED users tend to be more socially isolated and self-preoccupied than pro-recovery users, who tend to be more socially engaged than the first. Since members within a community tend to be more alike [52], there may not have been enough variation to identify associations with clinical variables in the present sample. Furthermore, given previous evidence that pro-recovery individuals sought positive emotions and expression of their true selves [5, 41], it was surprising that neither readiness to change nor ED severity predicted the Positive use factor. However, the present finding that enjoyment was the most endorsed motive overall suggests that this is likely to be a universal motive not related to individual differences relating to EDs [40]. Indeed, Alhabash and Ma [2] found that enjoyment was consistently the strongest predictor of use intensity of Facebook, Snapchat, Instagram, and Twitter, among a student sample.

With regard to our fourth hypothesis, stating that a lower readiness to change and more severe ED symptomatology would be associated with higher scores on the SNS use motives of reputation management and negative use (H4; [36], we could argue that the motives domains of Impression Management and Passive Use fit the description of motives in H4. The only motives dimension that was related to readiness to change was ‘Impression Management’ (i.e. social pressure, self-presentation, and popularity motives). More specifically, we found that individuals who used SNS to promote themselves, that is, wanted to be more popular or used it because they felt social pressure, were also less ready to change their unhealthy eating disordered behaviours. This latter may be very related to the finding that those who invested more in their online photos (i.e. posting and editing selfies) [7, 54] showed more eating disorder psychopathology than those who did not. Posting and editing selfies might be considered a form of online impression management. Results suggested that the lower the readiness to change, but the higher the body satisfaction, the more likely the participants were to use SNS for the purposes of impression management. Given that the present study found a positive correlation between body satisfaction and readiness to change (see Additional file 2: Table 2), this appears to be counterintuitive. However, it is possible that those who are more satisfied with their bodies would be more likely to show themselves for the purpose of recognition by others. Indeed, a recent study showed that higher body satisfaction was associated with greater Instagram selfie-posting in a sample of young women [7]. Here, to further elucidate these assumptions, it is important to study such reciprocal relations of how motives for social media use and ED symptomatology mutually affect each other in future longitudinal studies. The clinical implication is that using SNSs for impression management purposes could potentially hinder treatment and/or recovery of ED patients.

To continue on the dimension of Impression Management as an underlying motive for SNS use, age emerged as a significant negative predictor of the impression management motive dimension. Although age was not one of the specific variables of interest in this study, findings here hold important implications for the clinical practice. That is, the younger the participants, the more likely they were to be motivated by creating an identity for the purposes of popularity and social expectations. Young individuals are in the process of developing their identities and are therefore more likely to be concerned about others’ perceptions [6]. This is an important additional finding suggesting that clinicians and parents may need to pay special attention to young SNS users’ motives, rather than their total screen time.

None of the clinical variables of interest were significant contributors to the model predicting using SNSs for the purposes of passing time and avoiding negative emotions (the Passive Use factor). Although we have not specifically measured ritualistic and compulsive use as underlying motives, in further reasoning from these findings in the present study, one could argue that the results are not overly supportive of Perloff’s [36] idea that more vulnerable people (i.e., people with lower self-esteem, lower body satisfaction) are more likely to use SNSs repetitively and spontaneously to seek reassurance, procrastinate, escape from their problems and to reduce loneliness. Instead, from the high overall endorsement of escapism, avoiding loneliness, surveillance, and habitual use, our findings rather seem to suggest that these reasons may be common motives unrelated to ED symptoms. Indeed, Whiting and Williams [53] found that passing time and relieving boredom were important motives among their sample from the general population, with many of their respondents reporting that SNSs help them escape from reality and get away from daily stresses. This suggests that these may be universal SNS motives.

## Strengths, limitations and future research

As goes for all studies, our study has some strengths and limitations that can be considered, also in light of recommendations for future studies. First, we consider it a strength that we included participants with (current or past) clinical levels of eating disorder symptomatology, thereby addressing a vulnerable target group that is understudied in social media effects research. Furthermore, we form an interdisciplinary research team and combine principles from media psychology and communication science with the expertise of researchers within the ED-field.

This study also had some limitations, and as such, results should be interpreted with caution. First, no conclusions can be made about causality because the variables were measured simultaneously in a cross-sectional design. Future studies could employ longitudinal designs to investigate whether readiness to change predicts improvement in ED symptoms, or vice versa. The data collection started a few weeks after the start of the COVID-19 pandemic. At the time we were not yet fully aware of the impact. No variables were specifically included to measure stress regarding the pandemic. It is possible that more time at home due to lockdown, and less physical interaction with friends, could also have led participants to spend more time using SNSs, irrespective of ED severity [47]. Furthermore, the relatively small sample size may limit the interpretability of the results, especially given that results of factor analyses may not be reliable with samples below 300 [13]. Nevertheless, the current study found meaningful motives dimensions in line with other studies. Next, the completion rate of the survey was only 61% of those who initially signed the informed consent. However, there were no significant demographic differences between completers and non-completers (apart from a higher likelihood of living alone among non-completers) which suggests that the results were not influenced by demographic differences between those who provided complete data and those who did not. Although the survey was available for all genders, 97% of participants were female. This was not surprising, given that EDs are more prevalent in females than males.

Regarding the measurements we used, there is no standardized uses and gratifications questionnaire applicable to all SNS platforms, and there is no validated Dutch translation of the Readiness Ruler. Finally, the measure of SNS use (total time as a sum of the time spent on the different platforms) may have been the reason for the non-significant association between SNS use and ED psychopathology. The SNS landscape is continually increasing in complexity. It is possible to tweet one minute, and receive a Snapchat at the same time, all the while watching a YouTube video, and being logged into several other SNS platforms simultaneously. Future studies could obtain objective data (time logged in, content accessed) from individual SNS accounts to measure SNS activity more reliably and accurately.

## Conclusion

The objective of the present study was to examine the relationships between SNS use motives, ED symptom severity, and readiness to change ED behaviours and/or cognitions. The results suggest that if a person feels capable of overcoming their dysfunctional cognitions/behaviours about their body/eating, the less likely they are to use SNSs for impression management purposes, irrespective of the extent of their ED symptomatology. The clinical implication of this study is that being motivated by popularity, self-presentation, and social expectations may potentially hinder ED recovery. Clinicians may want to discuss motives of SNS use with their clients to help prevent any negative media effects.

### Supplementary Information

**Additional file 1. Table S1:** Motives Derived from Uses & Gratifications Studies.

**Additional file 2. Table S2:** Correlation Matrix dependent variables.

**Additional file 3. Table 3:** Correlation Matrix (Motives).

**Additional file 4. Table S4:** Exploratory Factor Analysis with Promax Rotation.

**Additional file 5. Table S5:** Tolerance and VIF Values for the dependent variables.

**Additional file 6. Table 6:** Durbin-Watson Values for Each Regression Analysis.

## Data Availability

The dataset used and analyzed during the current study are available from the corresponding author upon reasonable request.

## Footnotes

1 For example, the factor score for the factor Positive Use was computed by obtaining the mean from all the items that were used to measure enjoyment, relaxation, self-expression, and self-documentation.
