# Supplementary material for: Motives for using social networking sites: a uses & gratifications perspective amongst people with eating disorder symptoms
Source: J Eat Disord. 2023 Dec 19;11:231. doi: 10.1186/s40337-023-00946-1 (PMC10731897; doi:10.1186/s40337-023-00946-1)
Supplement: Supplementary file 1 — Additional file 1. Table S1: Motives Derived from Uses & Gratifications Studies. [file 40337_2023_946_MOESM1_ESM.docx]

Supplemental Table 1: Motives Derived from Uses & Gratifications Studies

| **The reason why I use SNS is…** | **GRATIFICATION** | **Source** |
| --- | --- | --- |
| Because it's fun | ENJOYMENT | Sheldon & Bryant (2016) |
| Because it’s enjoyable. | ENJOYMENT | Papacharissi & Mendelson (2011) |
| Because it’s entertaining. | ENJOYMENT | Papacharissi & Mendelson (2011) |
| Because it is soothing | RELAXATION | Bij de Vaate et al. (2018) |
| Because it relaxes me. | RELAXATION | Papacharissi & Mendelson (2011) |
| Because it is a pleasant rest. | RELAXATION | Papacharissi & Mendelson (2011) |
| Because it allows me to unwind. | RELAXATION | Papacharissi & Mendelson (2011) |
| To provide information | INFORMATION-SHARING | Papacharissi & Mendelson (2011) |
| To present information about a special interest of mine | INFORMATION-SHARING | Papacharissi & Mendelson (2011) |
| To share information that may be of use or interest to others | INFORMATION-SHARING | Papacharissi & Mendelson (2011) |
| To forget about school, work, or other things | ESCAPISM | Papacharissi & Mendelson (2011) |
| So I can get away from the rest of my family or others | ESCAPISM | Papacharissi & Mendelson (2011) |
| So I can get away from what I’m doing. | ESCAPISM | Papacharissi & Mendelson (2011) |
| So I won’t have to be alone. | AVOIDING LONELINESS | Papacharissi & Mendelson (2011) |
| When there’s no one else to talk or be with. | AVOIDING LONELINESS | Papacharissi & Mendelson (2011) |
| It makes me feel less lonely. | AVOIDING LONELINESS | Papacharissi & Mendelson (2011) |
| To communicate with distanced friends | SOCIAL INTERACTION | Papacharissi & Mendelson (2011) |
| To keep in touch with friends and family. | SOCIAL INTERACTION | Papacharissi & Mendelson (2011) |
| To connect with people I know | SOCIAL INTERACTION | Kim & Kim (2019) |
| To keep in touch with people I do not have enough time to see in person | SOCIAL INTERACTION | Kim & Kim (2019) |
| To keep connected with people who I otherwise would have lost contact with | SOCIAL INTERACTION | Chung (2014) |
| To find out what old friends are doing now | SOCIAL INTERACTION | Chung (2014) |
| To deepen relationships with people that I have met offline | SOCIAL INTERACTION | Chung (2014) |
| To keep in touch with people who live far away | SOCIAL INTERACTION | Chung (2014) |
| It is a habit, just something I do | PASSING TIME | Papacharissi & Mendelson (2011) |
| Because I have nothing better to do. | PASSING TIME | Papacharissi & Mendelson (2011) |
| Because it passes the time when I’m bored. | PASSING TIME | Papacharissi & Mendelson (2011) |
| It gives me something to do to occupy my time. | PASSING TIME | Papacharissi & Mendelson (2011) |
| To put off something I should be doing | PASSING TIME | Kim & Kim (2019) |
| To help others | COMMUNITY | Chung (2014) |
| To provide support to others | COMMUNITY | Chung (2014) |
| To contribute to discussions | COMMUNITY | Chung (2014) |
| To show others encouragement | COMMUNITY | Chung (2014) |
| To make new friends with similar interests | SIMILAR OTHERS | Chung (2014) |
| To meet new people with similar interests | SIMILAR OTHERS | Chung (2014) |
| To get to know other people | SIMILAR OTHERS | Chung (2014) |
| To keep in touch with people I have met via social media | SIMILAR OTHERS | Chung (2014) |
| To find people like me | SIMILAR OTHERS | Chung (2014) |
| To communicate with like-minded people | SIMILAR OTHERS | Chung (2014) |
| To connect with people who share some of my values | SIMILAR OTHERS | Alhabash & Ma (2017) |
| To gather information | SEEKING INFORMATION | Chung (2014) |
| To find out things that I need to know | SEEKING INFORMATION | Chung (2014) |
| To look for information I need | SEEKING INFORMATION | Chung (2014) |
| To talk to a knowledgeable individual about my issues | SEEKING INFORMATION | Chung (2014) |
| To get answers to specific questions | SEEKING INFORMATION | Chung (2014) |
| Because everybody else is doing it | SOCIAL PRESSURE | Papacharissi & Mendelson (2011) |
| Because it is the thing to do | SOCIAL PRESSURE | Papacharissi & Mendelson (2011) |
| Because it is cool | SOCIAL PRESSURE | Papacharissi & Mendelson (2011) |
| Because I feel social pressure to use it | SOCIAL PRESSURE | Bij de Vaate et al. (2018) |
| Because it is expected of me | SOCIAL PRESSURE | Bij de Vaate et al. (2018) |
| Because everybody does it | SOCIAL PRESSURE | Bij de Vaate et al. (2018) |
| To self-promote | POPULARITY | Sheldon & Bryant (2016) |
| To make me more popular | POPULARITY | Kim & Kim (2019) |
| To enhance my personal reputation | POPULARITY | Kim & Kim (2019) |
| To feel important | POPULARITY | Bij de Vaate et al. (2018) |
| To get recognition from other users | POPULARITY | Kim & Kim (2019) |
| To peek at others’ daily lives | SURVEILLANCE | Kim & Kim (2019) |
| Because I wonder what others do | SURVEILLANCE | Kim & Kim (2019) |
| To see what other people are doing | SURVEILLANCE | Kim & Kim (2019) |
| To see other people's updates | SURVEILLANCE | Sheldon & Bryant (2016) |
| To follow people | SURVEILLANCE | Sheldon & Bryant (2016) |
| To see what other people share. | SURVEILLANCE | Sheldon & Bryant (2016) |
| To creep through other people's posts/pages/photos | SURVEILLANCE | Sheldon & Bryant (2016) |
| To show my personality | SELF-EXPRESSION | Alhabash & Ma (2017) |
| To tell others about myself | SELF-EXPRESSION | Papacharissi & Mendelson (2011) |
| To show my creativity | SELF-EXPRESSION | Bij de Vaate et al. (2018) |
| To show who I am and what I do | SELF-EXPRESSION | Bij de Vaate et al. (2018) |
| To provide personal information about myself. | SELF-EXPRESSION | Papacharissi & Mendelson (2011) |
| To record what I do in life | SELF-DOCUMENTATION | Alhabash & Ma (2017) |
| To record what I have learned | SELF-DOCUMENTATION | Alhabash & Ma (2017) |
| To record where I have been | SELF-DOCUMENTATION | Alhabash & Ma (2017) |
| To share my life with other people | SELF-DOCUMENTATION | Sheldon & Bryant (2016) |
| To try out new identities | SELF-PRESENTATION | Bij de Vaate et al. (2018) |
| To present myself as I want others to see me | SELF-PRESENTATION | Bij de Vaate et al. (2018) |
| To show a different identity | SELF-PRESENTATION | Bij de Vaate et al. (2018) |
