# Supplementary material for: Motives for using social networking sites: a uses & gratifications perspective amongst people with eating disorder symptoms
Source: J Eat Disord. 2023 Dec 19;11:231. doi: 10.1186/s40337-023-00946-1 (PMC10731897; doi:10.1186/s40337-023-00946-1)
Supplement: Supplementary file 2 — Additional file 2. Table S2: Correlation Matrix dependent variables. [file 40337_2023_946_MOESM2_ESM.docx]

Supplemental Table 2: Correlation Matrix dependent variables

| **Variable** | Age | Readiness to Change | Body Satisfaction | Global EDE-Q | Self-Esteem | SNS Use |
| --- | --- | --- | --- | --- | --- | --- |
| Age | ~ |  |  |  |  |  |
| Readiness to Change | -.014 | ~ |  |  |  |  |
| Body Satisfaction | -.159 | .503** | ~ |  |  |  |
| Global EDE-Q | .076 | -.466** | -.715** | ~ |  |  |
| Self-Esteem | -.027 | .181 | .468** | -.464** | ~ |  |
| SNS Use | -.259** | .01 | -.038 | -.065 | -.045 | ~ |

*Note.* *p* < .05*, *p* < .01**; *n* = 114 for age, readiness to change, body satisfaction, EDE-Q, self-esteem; *n* = 103 for all correlations with SNS use. EDE-Q= Eating Disorder Examination-Questionnaire; SNS= social networking site
