# Supplementary material for: Motives for using social networking sites: a uses & gratifications perspective amongst people with eating disorder symptoms
Source: J Eat Disord. 2023 Dec 19;11:231. doi: 10.1186/s40337-023-00946-1 (PMC10731897; doi:10.1186/s40337-023-00946-1)
Supplement: Supplementary file 3 — Additional file 3. Table 3: Correlation Matrix (Motives). [file 40337_2023_946_MOESM3_ESM.docx]

Supplemental Table 3: Correlation Matrix (Motives)

|  | I-Sh | I-Se | S-D | S-E | S-P | Po | Pe | PT | Es | SI | AL | SPr | CSO | CS | En | Re |
| --- | --- | --- | --- | --- | --- | --- | --- | --- | --- | --- | --- | --- | --- | --- | --- | --- |
| Information-Sharing | ~ |  |  |  |  |  |  |  |  |  |  |  |  |  |  |  |
| Information-Seeking | .18 |  |  |  |  |  |  |  |  |  |  |  |  |  |  |  |
| Self-Documentation | .31 | .15 |  |  |  |  |  |  |  |  |  |  |  |  |  |  |
| Self-Expression | .55 | .10 | .74 |  |  |  |  |  |  |  |  |  |  |  |  |  |
| Self-Presentation | .14 | .03 | .35 | .45 |  |  |  |  |  |  |  |  |  |  |  |  |
| Popularity | .22 | .00 | .39 | .45 | .75 |  |  |  |  |  |  |  |  |  |  |  |
| Peeking | .20 | .25 | .23 | .29 | .21 | .18 |  |  |  |  |  |  |  |  |  |  |
| Passing Time | .00 | .27 | .18 | .20 | .22 | .14 | .45 |  |  |  |  |  |  |  |  |  |
| Escapism | .10 | .18 | .11 | .13 | .24 | .15 | .24 | .45 |  |  |  |  |  |  |  |  |
| Social Interaction | .24 | .21 | .47 | .47 | .30 | .34 | .42 | .23 | .07 |  |  |  |  |  |  |  |
| Avoiding Loneliness | .30 | .28 | .26 | .35 | .35 | .23 | .30 | .46 | .48 | .22 |  |  |  |  |  |  |
| Social Pressure | .05 | .06 | .36 | .24 | .35 | .32 | .17 | .08 | .12 | .28 | .28 |  |  |  |  |  |
| Connecting with Similar Others | .47 | .19 | .38 | .42 | .22 | .21 | .09 | -.04 | .04 | .22 | .39 | .22 |  |  |  |  |
| Community of Support | .54 | .24 | .39 | .42 | .24 | .22 | .13 | -.03 | .03 | .35 | .32 | .31 | .62 |  |  |  |
| Enjoyment | .09 | .09 | .31 | .25 | .06 | .15 | .22 | .18 | -.03 | .11 | .08 | -.05 | .04 | -.02 |  |  |
| Relaxation | .05 | .05 | .31 | .21 | .04 | .10 | .14 | .16 | .08 | .22 | .04 | -.06 | .08 | .10 | .31 | ~ |
