# Supplementary material for: Motives for using social networking sites: a uses & gratifications perspective amongst people with eating disorder symptoms
Source: J Eat Disord. 2023 Dec 19;11:231. doi: 10.1186/s40337-023-00946-1 (PMC10731897; doi:10.1186/s40337-023-00946-1)
Supplement: Supplementary file 4 — Additional file 4. Table S4: Exploratory Factor Analysis with Promax Rotation. [file 40337_2023_946_MOESM4_ESM.docx]

Supplemental Table 4: Exploratory Factor Analysis with Promax Rotation

|  | **Community** | **Impression Management** | **Habit** | **Authenticity** |
| --- | --- | --- | --- | --- |
| Information-Sharing | .66 |  |  |  |
| Information-Seeking |  |  |  |  |
| Self-Documentation |  |  |  | .57 |
| Self-Expression |  |  |  | .46 |
| Self-Presentation |  | .98 |  |  |
| Popularity |  | .87 |  |  |
| Surveillance |  |  | .42 |  |
| Passing Time |  |  | .79 |  |
| Escapism |  |  | .61 |  |
| Social Interaction |  |  |  |  |
| Avoiding Loneliness |  |  | .65 |  |
| Social Pressure |  | .40 |  |  |
| Connecting with Similar Others | .80 |  |  |  |
| Support | .88 |  |  |  |
| Enjoyment |  |  |  | .60 |
| Relaxation |  |  |  | .54 |
|  |  |  |  |  |
| **Cronbach’s alpha** | .92 | .90 | .91 | .90 |
| **Eigenvalue** | 4.71 | 1.9 | 1.53 | 1.51 |
| **% of Variance** | 29.46 | 11.9 | 9.56 | 9.4 |

*Note:* Loadings <.4 not included in this table.
