# Supplementary material for: Motives for using social networking sites: a uses & gratifications perspective amongst people with eating disorder symptoms
Source: J Eat Disord. 2023 Dec 19;11:231. doi: 10.1186/s40337-023-00946-1 (PMC10731897; doi:10.1186/s40337-023-00946-1)
Supplement: Supplementary file 5 — Additional file 5. Table S5: Tolerance and VIF Values for the dependent variables. [file 40337_2023_946_MOESM5_ESM.docx]

Supplemental Table 5: Tolerance and VIF Values for the dependent variables

|  | **Tolerance** | **VIF** |
| --- | --- | --- |
| Age | 0.94 | 1.01 |
| Global EDE-Q | 0.43 | 2.34 |
| Body Satisfaction | 0.46 | 2.18 |
| Self-Esteem | 0.70 | 1.44 |
| Readiness to Change | 0.66 | 1.52 |
| SNS Use | 0.93 | 1.08 |
