# Supplementary material for: Motives for using social networking sites: a uses & gratifications perspective amongst people with eating disorder symptoms
Source: J Eat Disord. 2023 Dec 19;11:231. doi: 10.1186/s40337-023-00946-1 (PMC10731897; doi:10.1186/s40337-023-00946-1)
Supplement: Supplementary file 6 — Additional file 6. Table 6: Durbin-Watson Values for Each Regression Analysis. [file 40337_2023_946_MOESM6_ESM.docx]

Supplemental Table 6: Durbin-Watson Values for Each Regression Analysis

| **Regression** | **Outcome Variable** | **Durbin-Watson** |
| --- | --- | --- |
| 1 | Community | 2.2 |
| 2 | Impression Management | 1.86 |
| 3 | Passive Use | 1.82 |
| 4 | Positive Use | 1.91 |
